# Impact of Coordinated-Bilateral Physical Activities on Attention and Concentration in School-Aged Children

**DOI:** 10.1155/2018/2539748

**Published:** 2018-05-28

**Authors:** Heidi Buchele Harris, Kai Schnabel Cortina, Thomas Templin, Natalie Colabianchi, Weiyun Chen

**Affiliations:** ^1^School of Kinesiology, University of Michigan, Ann Arbor, MI 48109, USA; ^2^Department of Psychology, University of Michigan, Ann Arbor, MI 48109, USA

## Abstract

**Purpose:**

This study examined the effects of 4-week, daily 6-minute coordinated-bilateral physical activity (CBPA) breaks in classroom on attention and concentration in school-aged children.

**Methods:**

Participants (n=116) in fifth grade from two elementary schools were assigned to three groups: two intervention groups (n= 60) and one control group (n = 56). All three groups were pre- and post-tested with the d2 Test of Attention (d2 test). One intervention group (n = 31) took part in six minutes of daily classroom-based coordinated-bilateral physical activity (CBPA) break for four weeks. Another intervention group (n = 29), the Fitbit Only (Fitbit-O), wore Fitbits per day during a school, five days per week for four weeks without CBPA breaks. A 2 × 3 ANOVA was conducted, followed by the post hoc comparisons.

**Results:**

The CBPA showed significant increases in processing speed (*F*_1_ = 6.876,* p* = .010), focused attention (*F*_1_ = 10.688,* p* = .002), concentration performance (*F*_1_ = 26.46,* p* = .000), and attention span (*F*_1_ = 14.090,* p* = .000) over the control, but not in accuracy (Error %). The CBPA showed significant improvement in concentration performance (*F*_1_ = 24.162,* p* = .000) and attention span (*F*_1_ = 6.891,* p* = .011), compared to the Fitbit-O. No significant changes in all five attention parameters were found between the Fitbit-O and the control.

**Conclusion:**

It was concluded that daily brief coordinated-bilateral activities can improve attention and concentration in fifth-grade students over the course of four weeks.

## 1. Introduction

Studies have shown that physical activity (PA) is positively associated with academic performance [1–11]. Individuals are more likely to have better mental focus and concentration when engaging in structured physical activity [11–18]. However, children are sitting in the classroom for most of a school day [1–3]. A prolonged sedentary behavior not only does reduce children's attention to instructional tasks and their sustained focus on the task engagement, but also prevents children from meeting a recommended daily amount of moderate-to-vigorous physical activity (MVPA) [11–20]. To address the critical concerns, classroom-based PA breaks have been increasingly used as an effective intervention strategy to improve students' academic achievement, academic behaviors, and executive functions [11–20].

A handful studies have examined the impact of classroom-based PA breaks on attention and concentration quantitatively and qualitatively [3, 19–28]. The studies have shown that the classroom-based PA can improve children's attention and concentration [19–24]. In a study of implementing daily 10-minute physically active academic lessons (Energizers) to 62 third- and four-grade students for 12 weeks, Mahar et al. [23] found significant increases in on-task behaviors (i.e., appropriate verbal or motor behaviors) from pre-Energizers treatment to post-Energizers treatment. Additionally, students in the energizer group took a significantly higher number of steps than the control group by the end of each day [23]. Similarly, in a study of implementing a daily 10-15 minutes physically active lesson (Texas I-CAN) to third-grade students, researchers observed each student's time-on-task (TOT) within 15-minute before and after the physically active lesson and the inactive lesson (control) for 16-22 times [24]. The study found that the students' TOT was increased slightly from before to after the active lesson, while the students' TOT was decreased significantly from before to after the inactive lesson.

Although classroom-based PA studies have documented a willingness of teachers to use physically active lessons, some barriers such as added training, extra preparation time, and additional demanding on knowledge base for implementing physically active lessons may lead to difficulties for scalable and sustainable implementation [3, 22, 25]. To reduce additional workload for the teacher to implement the classroom-based PA breaks, Howie et al. [26] examined how long the classroom PA breaks should last using a four-tiered approach of 10-minute sedentary breaks versus 5, 10, and 20 minutes of classroom-based PA breaks. They found that all PA breaks showed statistically significant improvements in time-on-task (TOT) with the most pronounced effect occurring with the 10-minute PA breaks compared to the sedentary break. Further, Ma et al. [27] found that four minutes of in-class high-intensity PA breaks increased selective attention and reduced errors assessed with d2 Test of Attention in 9- to 11-year old children.

More recently, researchers have examined the effects of coordinated-bilateral exercises on attention and concentration in school-aged children [2, 28]. Coordinated-bilateral exercises are specifically designed to use both sides of the body and more body parts simultaneously to perform bilateral movements while crossing the midline of the body [2, 28]. Coordinated-bilateral movements engage both hemispheres of the brain and may facilitate cognitive development of cerebellum and prefrontal cortex [29, 30]. Budde et al. [2] found that the coordinated-bilateral exercise in physical education (PE) lessons led to significant improvements in children's attention assessed with d2 Test of Attention. Similarly, Schmidt et al. [28] examined 90 fifth-grade students' attention performance tested with the revised version of the d2 Test of Attention (d2-R) before, immediately after, and 90 minutes after an acute bout of coordinative exercise in PE. They found that the coordinated exercise significantly increased children's attentional performance 90 min. after cessation, but not immediate. Furthermore, in a systematic review of PA interventions, Van der Fels et al. [31] suggest that the short burst of fine and gross motor coordinated-bilateral PA can improve attention, processing speed, and focus [31]. However, the previous studies have limited to examining one time effects of coordinated-bilateral exercise on attention and concentration, the key component of executive functions. Therefore, this study aimed at examining the effect of 4-week, daily 6-minute coordinated-bilateral physical activity (CBPA) breaks after 20 minutes of sedentary on attention and concentration in school-aged children. This study hypothesized that students in the CBPA breaks would show a greater increase in their attentional performance measured with the d2 Test of Attention compared to the comparison groups. The significance of this study was that the intervention was designed for the classroom teachers to instantly engage all students in CBPA breaks without any additional preparation by showing 6-minute CBPA videos. Therefore, the study intervention strategy would be feasible and scalable for classroom teachers to implement.

## 2. Methods

### 2.1. Design

This study used a quasi experimental design to assign one elementary school to the intervention school and another to the control school. In the intervention school, one fifth-grade class was assigned to the CBPA group (n=31, 17 boys versus 14 girls), and another fifth-grade class was assigned to Fitbit Only (Fitbit-O) group (n=29, 19 boys versus 10 girls). In the control school, two fifth-grade classes were assigned to the comparison group (n=56, 21 boys versus 35 girls). This study was conducted over the course of seven weeks. The first week was used for recruitment. The students took the d2 Test of Attention at baseline during the second week and took the test again immediately after the 4-week intervention during the seventh week.

### 2.2. Participants

Approval was obtained from the University Institutional Review Board- (IRB-) Health Sciences and Behavioral Sciences (HSBS) (HUM00102732). The signed consent forms were obtained from the parent/guardian of 116 students who were recruited from four, fifth-grade classes at two elementary schools. Also, written assent forms were gathered from the students prior to pre- and post-testing. Two elementary schools with a racially and socioeconomically diverse student population were selected based on their fiscal and racial similarities and their voluntary participation in this study. 43% of students in the intervention school and 37% of students in the control school received free or reduced lunch. In the intervention school, 60% of the participants self-identified a race other than white, with 30% African American. At the comparison school, 48% self-identified a race other than white, with 19% African American.

### 2.3. Treatments

*Fitbits*. Students in the CBPA and the Fitbit-O groups were given Fitbits Charger HR to wear from Monday morning (as they arrived at the classroom) until Friday afternoon, whereupon they put the Fitbits in the charging station before they left for the weekend for four weeks, except in cases when they might get wet. Students were able to check their own steps taken, real time heart rate, distances traveled, and calorie burned on the Fitbit. Students were encouraged to keep track of their own information and to set goals for themselves, while teachers were given reports of the classroom average steps and activity minutes each Monday by the investigator. In contrast, the students in the control group wore plastic wristband for four weeks.

*Coordinated-Bilateral Physical Activity (CBPA)*. Students in the CBPA group participated in 6-minute repetitive coordinated-bilateral motor movements while following 6-minute video instructor's CBPA once a day after they had been sitting for 20 minutes of a class instruction for five days per week, over four weeks. The CBPA videos were deliberately designed to emphasize coordinated exercises which use bilateral body movements and to be progressive in difficulty and speed [32]. The CBPA videos started slowly and rhythmically during the first week and were gradually done faster and more complicated in weeks of 2 and 3. During the week 4, the levels of difficulty and speed were further increased. Motor movements were rhythmically repeated eight, four, and two first in unison and then in opposite directions. Examples of motor movements were making figure eights by simultaneously pairing arm movements in the same direction, by changing the direction, and by having the arms go in opposite directions. Then, motor skills utilized the entire body. Children went from a split to a squat stance, first in unison then in opposite directions, so the video instructor jumped sideways, and the participants were encouraged to squat. The teacher reported missing 3 days of CBPA. The Quick Time videos were labeled Day 1 through Day 20. The control group was not asked to make any changes to their normal school day beyond taking the additional pre- and post-d2 Tests administered to all three groups.

*Comparison Group. *The students in the comparison group followed their school scheduled academic instruction periods. In other words, the comparison group was not asked to make any changes to their normal school day beyond taking the additional pre- and post-d2 Tests of Attention, which were administered in all three groups at pre- and post-test data collection.

### 2.4. Data Collection

On October 21 students in the Fitbit-O and CBPA group and on October 26 students in the comparison group began the pre-test. Prior to the pre-test, the first author explained and showed how to take the d2 Test based on the standardized testing directions and then asked the student to practice the two lines of the test provided on the standardized test directions to ensure that all students understand the testing procedures. Once the students completed the d2 Test of Attention, students in the two intervention groups were given their Fitbits and were encouraged to wear them Monday through Friday for four weeks. On November 17 students in the Fitbit-O and CBPA group and on November 26 the students in the comparison group took the post-test while following the same testing procedures as the pre-test as well.

*D2 Test of Attention.* Prior to the beginning of this study, teachers were consulted about what they would like to see their students being tested. Teachers noted that minimizing the amount of time spent on testing and showing gains in attention and concentration are important factors to be considered. Accordingly, the d2 Test of Attention was chosen. It is easy for students to understand how to take the test given the standardized directions for the test. Also, students will take 4.67 minutes to complete the test. The d2 Test is a cancellation test that measures neuropsychology performance of the students in the areas of sustained and selective attention as well as concentration. The d2 Test allows participants 20 seconds per line to selectively identify the letter “d” with two dashes, either above, below, or one dash on top and one on the bottom. Distractors come in two forms, more or less dashes above or below the “d” and the letter “p” [33].

(1) $$\begin{array}{c} \begin{array}{ccc} \cdot \cdot & & \cdot \\ d & d & d\\ & \cdot \cdot & \cdot \end{array} \end{array}$$

The d2 Test offers five main outcomes (parameters): (a) the total number of items processed (TN) (i.e., processing speed); (b) the total number of symbols processed minus the total number of errors (TN-E) (i.e., focused attention); (c) the total number of correct responses minus commission errors (CP) (i.e., concentration performance); it measures the ability to attend to stimuli while disregarding other irrelevant tasks; (d) the percentage of all errors (E%), in which omission and commission are made within all items processed (i.e., accuracy); and (e) fluctuation rate (FR): it is determined by subtracting the line with the lowest number of symbols process from the line with the highest number of symbols process (i.e., sustained attention) [33]. The d2 Test had high test-retest reliability coefficients for all parameters, ranging from .95 to .98 [33]. The d2 Test has been proven to be an internally valid measure of scanning accuracy, speed, discriminant validity, and fluctuation across trials [34].

### 2.5. Data Analysis

TN, TN-E, CP, E%, and FR were used as parameters of attention and concentration performance for data analysis of the d2 Test based upon the guidelines set by Brickenkamp et al. [33]. Descriptive statistics of TN, TN-E, CP, E%, and FR at pre-test and post-test were conducted for each group. A 2×3 analysis of variance (ANOVA) was conducted with the time (pre-test versus post-test) as a within-subjects factor and treatment conditions (CBPA, Fitbit-O, and control) as a between-subjects factor. The repeated measure ANOVAs were conducted separately for the TN, TN-E, CP, E%, and FR. Subsequently, a post hoc comparison was performed to examine the mean difference in each dependent variable between groups (i.e., CBPA-Bs versus Fitbit-O; CBPA-Bs versus control; and Fitbit-O versus control) from pre- to post-test. All statistical analyses were conducted with IBM SPSS version 24 and a significant level of* p* < .05 was set.

## 3. Results

### 3.1. Preliminary Analysis

Table 1 presents the descriptive statistics of pre- and post-test in d2 Test of Attention among the three groups. At pre-test, the control group's mean scores in processing speed (TN), focused attention (TN-E), and concentration performance (CP) were higher than the two intervention groups. The higher numbers of TN, TN-E, and CP indicate the better performance in attention and concentration. The control group's mean score in accuracy (E%) was similar to the CBPA's mean score and lower than the Fitbit-O's mean score. The control group's mean score in attention span (FR) was similar to the Fitbit-O's mean score and lower than the CBPA group's mean score. The lower scores in E% and FR represent the better accuracy and attention span. Further, regarding the pre-test, independent sample t-tests revealed no significant mean differences between CBPA and Fitbit-O groups in TN, TN-E, CP, E%, and FR. Similarly, there is no significant mean difference in E% and FR between CBPA group and the control group. However, the t-tests showed that the control group exhibited significantly higher scores in TN, TN-E, and CP than the CBPA group (t = 4.058,* p* = .000; t = 3.571,* p* = .001; t = 2.944,* p* = .002).

Regarding the changes from pre- to post-test scores for each group, all three groups showed improvement in mean scores of TN, TN-E, CP, and E% compared to the pre-test. The CBPA group's mean score in FR decreased from the pre-test, while both Fitbit-O group's and the control group's mean score of FR increased compared to the pre-test. Across the three groups, the CBPA group's mean scores in TN-E and CP were higher and mean score in E% was lower than the two groups; the control group's mean scores in TN-E and CP were higher and mean score in E% was lower than the Fitbit-O group. However, the CBPA group's mean scores in TN and FR were lower than the control group, but higher than the Fitbit-O group.

### 3.2. Results of Repeated Measure ANOVA

Table 2 illustrates the results of the repeated measure ANOVA. The results showed significant main effect of time in processing speed (TN) (*F*_1_ = 140.52,* p* = .000, *η*^2^= 0.561), focused attention (TN-E) (*F*_1_ = 193.44,* p* = .000, *η*^2^= 0.637), concentration performance (CP) (*F*_1_ = 160.14,* p* = .000, *η*^2^= 0.593), and accuracy (E%) (*F*_1_ = 21.35,* p* = .000, *η*^2^= 0.163), but not in FR. The three groups showed significant improvement in processing speed, focused attention, concentration performance, and accuracy from pre- to post-test.

Further, as presented in Table 2, the repeated measure ANOVA revealed a significant interaction between time × treatment in processing speed (TN) (*F*_2_ = 3.372,* p* = .038, *η*^2^= 0.058), focused attention (TN-E) (*F*_2_ = 4.37,* p* = .015, *η*^2^= 0.074), concentration performance (CP) (*F*_2_ = 13.53, *p* = .000, *η*^2^ = 0.197), and attention span (FR) (*F*_2_ = 8.04,* p* = .001, *η*^2^= 0.128 ), but not in accuracy (E%). The results indicated that significant changes in processing speed, focused attention, concentration performance, and attention span were associated with within subject factors (from pre- to post-test) and between subject factors (three groups), but not related to accuracy.

### 3.3. Results of Post Hoc Comparisons

Table 3 shows the post hoc comparisons between two groups from pre- to post-test. The results indicated that there were significant differences in concentration performance (CP) (*F*_1_ = 24.162,* p* = .000) and attention span (FR) (*F*_1_ = 6.891,* p* = .011) between the CBPA and the Fitbit-O group from pre- to post-test, but not in TN, TN-E, and E%. This means that students in the CBPA were better able to concentrate and sustain their attention after the intervention compared to Fitbit-O group. Further, significant changes between the CBPA group and the control group were found in processing speed (TN) (*F*_1_ = 6.876,* p* = .010), focused attention (TN-E) (*F*_1_ = 10.688,* p* = .002), concentration performance (CP) (*F*_1_ = 26.46,* p* = .000), and attention span (FR) (*F*_1_ = 14.090,* p* = .000), but not in accuracy (E%). The results indicated that students in the CBPA significantly increased their scores in TN, TN-E, CP, and FR over the control group. In contrast, no significant changes in all five parameters were found between the Fitbit-O and the control groups.

## 4. Discussion

This study was central to examining whether students who participated in coordinated-bilateral PA breaks showed a greater increase from pre- to post-test in attention and concentration than students in the Fitbit-O and the control groups. In line with the results of previous studies [2, 28], the students in the CBPA group demonstrated a significantly greater increase from pre- to post-test on the d2 Test than the students in the Fitbit-O and the control groups. Largely supporting the hypothesis, the results indicated that the 4-week, daily 6-minute coordinated-bilateral PA breaks in a classroom significantly improved children's processing speed (TN), focused attention (TN-E), concentration performance (CP), and attention span (FR), compared to the Fitbit-O and the control groups.

The CBPA was designed to facilitate the exchange of information between the cerebellum and prefrontal cortex by focusing on the movement sequences [29, 30]. The CBPA was accomplished by using timed sequences, repetitive patterned movements, and contralateral movements to engage both hemispheres of the brain [28]. Further, the present results supported that “motor development and cognitive development may be fundamentally related” [29] (p.44). This study supported the notion that highly focused, coordinated-bilateral activities in short increments improved attention and concentration through motor development without the need to add an academic component [2, 29–31]. The CBPA was found to be a feasible alternative to academic lessons, while still providing a PA break for the classroom.

The uniqueness of this study was that the 4-week, daily 6-minute CBPA breaks showed pronounced improvement in processing speed (TN), focused attention (TN-E), concentration performance (CP), and attention span (FR). Previous studies examined the acute effect of a single bout of aerobic coordinated movement on attention assessed with d2 Test [2, 15, 28]. Budde et al. [2] found significant improvements in processing speed (TN), focused attention (TN-E), and accuracy (E%). But, they did not found improvement in concentration performance (CP) and attention span (FR). Schmidt et al. [28] reported significant increases in processing speed (TN), accuracy (E%), and concentration performance (CP), but not in focused attention (TN-E) and attention span (FR). This study indicated that the addition of short CBPA breaks into the classroom routine helped children sustain and sharpen their attention. By helping children find a way to better attend to their learning, schools may be able to reduce further problems of self-control and thus improve academic outcomes for all students [2, 28, 31].

Also, it is important to note that there were significant differences in concentration performance (CP) and attention span (FR) between the CBPA and Fitbit-O group, but no significant differences in the five parameters between the Fitbit-O and the control group. This adds merit to the idea that adding highly focused, coordinated-bilateral activities to the school day can bring about positive changes in concentration and sustained attention, but the non-focused PA may not. However, the results did reveal that the non-focused PA may potentially affect a student's processing speed (TN), focused attention (TN-E), and accuracy (E%). Supporting the result, Schmidt et al. [28] found the most significant improvement in attention and concentration came from students' engaging in highly aerobic activities. Similarly, Gallotta et al. [15] found improvements in attention and concentration with aerobic activity and any type of breaks, compared no breaks. Future studies may consider adding both highly focused, sequentially coordinated exercises and aerobic activities to academic lessons to see if there is a significant difference between these strategies.

This study highlights three reasons why using the CBPA strategies is a feasible option for classrooms. First, they do not require additional space. Unlike many of the other studies, this study did not use the gym, playground, or hallways. Instead, children were able to stand next to their desks to complete the activities. Secondly, no additional teachers' training in CBPA breaks or researchers were needed to facilitate the CBPA breaks. In contrast, other classroom-based PA breaks are required in-service training for the teachers [22, 23]. Finally, the interruption to the child's academic learning was minimal, only six minutes. In short, students who participated in the 4-week, daily 6-minute CBPA breaks without any additional academic component showed improvements in attention and concentration.

It is important to note that this study has four limitations. The first limitation was related to potential confounders (e.g., IQ and SES) that may influence the children's attentional performance. Although this study did not directly collect the information from the students, the participating students who enrolled in the two participating schools came from ethnic and racial diverse and low-income neighborhood. The second limitation is that the study was conducted in schools which made it difficult to do the random assignment. We used available schools as the participating schools (convenient samples) and assigned the schools to either the experimental or the control condition based on their preference. This may account for the higher baseline d2 scores found in the control school. The third limitation of the study which is related to the students' use of the Fitbit Challenge may also have impacted the outcomes for the d2 test, in addition to engaging in the 4-week, daily 6-minute CBPA breaks. However, the differences between the two intervention groups suggested that the Fitbit itself did not significantly alter the results. The likely increase in PA which was brought about by the Fitbit Challenge may account for the differences on the d2 Test between the CBPA group and the Fitbit-O group and the control group. Additionally, both intervention groups were meant to take part in the Fitbit Challenge, but the teacher from the Fitbit-O group opted out of this part of the study. Therefore, the impact of the coordinated-bilateral activities cannot be said to be solely responsible for the differences in the d2 Test. The last limitation was associated with no emphasis placed on increasing aerobic activity in this intervention. So, there was no direct comparison which could be made between aerobic and coordinated-bilateral activities. Future studies may add aerobic-typed PA to the coordinated-bilateral activities for running the classroom-based PA breaks.

It was concluded that students who participated in 4-week, daily 6-minute CBPA breaks showed significant improvements in processing speed (TN), focused attention (TN-E), concentration performance (CP), and attention span (FR), compared to the control group. The unique result was that the 4-week, daily highly focused, coordinated-bilateral activities showed the significant intervention effects on improving concentration performance and attention span, especially compared to the students who merely focused on increasing their PA levels. This study suggests that engaging students in daily, highly focused, coordinated-bilateral activities is an effective strategy to improve attention and concentration in school-aged children.

## Figures and Tables

**Table 1:** Descriptive statistics of pre- and posttest among CBPA, Fitbit-O, and control groups.

	Pretest M ± SD	Posttest M ± SD
CBPA (n=31)		
Processing Speed (TN)	271.68±38.99	362.65±76.27
Focused Attention (TN-E)	250.84±41.81	353.03±77.16
Concentration Performance (CP)	93.65±26.57	138.68±29.68
Accuracy (Error %)	7.15±7.72	2.74±3.55
Attention Span (FR)	17.32±7.02	14.39±7.19

Fitbit-O (n=28)		
Processing Speed (TN)	278.79±49.46	357.14±73.36
Focused Attention (TN-E)	257.29±48.28	336.86±67.66
Concentration Performance (CP)	94.11±25.23	109.93±33.95
Accuracy (Error %)	8.96±9.06	6.47±9.24
Attention Span (FR)	14.86±5.82	18.90±9.89

Control (n=55)		
Processing Speed (TN)	317.7±66.37	374.18±82.18
Focused Attention (TN-E)	293.02±61.67	350.55±82.83
Concentration Performance (CP)	112.58±34.04	133.00±34.95
Accuracy (Error %)	7.19±10.01	4.34±7.83
Attention Span (FR)	14.77±7.27	17.87±8.54

**Table 2:** Results of the repeated measure ANOVA.

Dependent Variables	Factors	F	df	P	*η* ^2^
Processing Speed (TN)	Time	140.52	1	.000	.561
	Time *∗* Treatment	3.372	2	.038	.058
Focused Attention (TN-E)	Time	193.44	1	.000	.637
	Time *∗* Treatment	4.37	2	.015	.074
Concentration Performance (CP)	Time	160.14	1	.000	.593
	Time *∗* Treatment	13.53	2	.000	.197
Accuracy (Error %)	Time	21.35	1	.000	.163
	Time *∗* Treatment	.194	2	.824	.004
Attention Span (FR)	Time	2.71	1	.102	024
	Time *∗* Treatment	8.04	2	.001	.024

**Table 3:** Results of post hoc comparisons between groups in five dependent variables.

Dependent Variables	Between Groups	*F*	*df*	*P*
Processing Speed (TN)	CBPA vs. Fitbit-O	.925	1	.340
	CBPA vs. Control	6.876	1	.010
	Fitbit-O vs. Control	1.842	1	.178

Focused Attention (TN-E)	CBPA vs. Fitbit-O	1.73	1	.194
	CBPA vs. Control	10.688	1	.002
	Fitbit-O vs. Control	1.874	1	.175

Concentration performance (CP)	CBPA vs. Fitbit-O	24.162	1	.000
	CBPA vs. Control	26.454	1	.000
	Fitbit-O vs. Control	.001	1	.976

Accuracy (Error %)	CBPA vs. Fitbit-O	.538	1	.466
	CBPA vs. Control	.297	1	.587
	Fitbit-O vs. Control	.001	1	.976

Attention Span (FR)	CBPA vs. Fitbit-O	6.891	1	.011
	CBPA vs. Control	14.090	1	.000
	Fitbit-O vs. Control	0.568	1	.453
